# Quintuple Primary Cancers Including Intraductal Papillary Mucinous Carcinoma: A Case Report

**DOI:** 10.70352/scrj.cr.26-0046

**Published:** 2026-04-14

**Authors:** Taiki Mori, Daisuke Suzuki, Tsukasa Takayashiki, Shigetsugu Takano, Nozomu Sakai, Isamu Hosokawa, Takashi Mishima, Takanori Konishi, Kensuke Suzuki, Hitoe Nishino, Shinichiro Nakada, Masayuki Ohtsuka

**Affiliations:** 1 Department of General Surgery, Graduate School of Medicine, Chiba University, Chiba, Chiba, Japan

**Keywords:** IPMN, IPMC, multiple primary malignancies, extrapancreatic malignancies, surveillance

## Abstract

**INTRODUCTION:**

The incidence of multiple primary malignancies has increased owing to advances in cancer screening and treatment. However, cases involving five distinct primary cancers remain extremely rare. Intraductal papillary mucinous neoplasms (IPMNs) are known to be associated with extrapancreatic malignancies. We report a very uncommon case of quintuple primary cancers, including noninvasive intraductal papillary mucinous carcinoma (IPMC), and discuss its clinical implications.

**CASE PRESENTATION:**

A 77-year-old man was referred for evaluation of pancreatic duct dilatation incidentally detected during staging for hypopharyngeal cancer. He had been diagnosed with hypopharyngeal squamous cell carcinoma and underwent radiotherapy. During this evaluation, a colonoscopy revealed sigmoid colon cancer, which was treated with a laparoscopic sigmoidectomy (pT2N0M0). Imaging revealed main pancreatic duct dilatation with a 15-mm enhancing mural nodule in the pancreatic head, meeting the criteria for high-risk stigmata of IPMN and prompting further pancreatic evaluation. Endoscopic ultrasonography demonstrated a 19 × 4 mm intraductal papillary lesion in the pancreatic head duct, consistent with an IPMN with high-risk stigmata. A pancreaticoduodenectomy was performed, and the pathology report confirmed noninvasive IPMC with negative margins and nodes (pTisN0M0). One year later, routine surveillance detected a solitary right middle-lobe lung nodule; thoracoscopic lobectomy confirmed squamous cell carcinoma (pT1aN0M0). At age 81, the patient was diagnosed with prostate cancer and began hormonal therapy. Histopathological diagnoses revealed five distinct malignancies without evidence of metastasis. The patient is currently alive and under surveillance without evidence of recurrence.

**CONCLUSIONS:**

Quintuple primary cancers including noninvasive IPMC are very uncommon. Patients with IPMN/IPMC may require risk-adapted and long-term follow-up for synchronous or metachronous malignancies.

## Abbreviations

CA19-9
: carbohydrate antigen 19-9
CEA
: carcinoembryonic antigen
EUS
: endoscopic ultrasonography
FDG
: fluorodeoxyglucose
IHC
: immunohistochemistry
IPMC
: intraductal papillary mucinous carcinoma
IPMN
: intraductal papillary mucinous neoplasm
MMR
: mismatch repair
MPM
: multiple primary malignancy
MSI
: microsatellite instability
MSS
: microsatellite stable
PSA
: prostate-specific antigen
RT
: radiotherapy

## INTRODUCTION

**Fig. 1:**
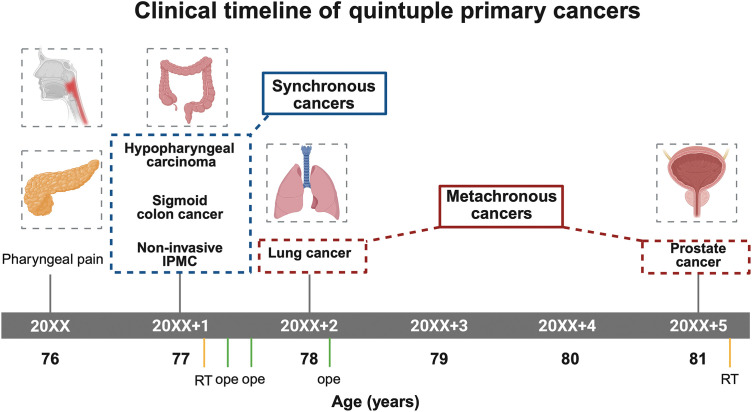
Clinical timeline of quintuple primary cancers. Synchronous cancers including hypopharyngeal carcinoma, sigmoid colon cancer, and noninvasive IPMC were diagnosed at age 77, followed by lung cancer at age 78 and prostate cancer at age 81. IPMC, intraductal papillary mucinous carcinoma; RT, radiotherapy

Multiple primary malignancies (MPMs) are defined as two or more distinct malignant tumors that are not metastases of one another.^1)^ Their incidence is increasing due to aging populations and improved diagnostics. Among pancreatic cystic lesions, IPMNs are well recognized for their association with synchronous or metachronous extrapancreatic cancers, which has been reported in 15%–30% of cases.^2,3)^ Although several authors have described cases of double or triple primaries accompanying IPMN, quintuple malignancies are exceedingly uncommon. Here, we present the case of an elderly man who sequentially developed hypopharyngeal squamous cell carcinoma, noninvasive IPMC, sigmoid colon adenocarcinoma, lung squamous cell carcinoma, and prostate adenocarcinoma (**Fig. 1**). Reports of quintuple primary cancers including noninvasive IPMC are scarce. We also discuss the diagnostic challenges, potential pathogenetic links, and surveillance implications.

## CASE PRESENTATION

A 77-year-old Japanese man presented with a 2-month history of pharyngeal discomfort. He had a smoking history of 40 cigarettes/day for 23 years (approximately 46 pack-years) and quit smoking at age 43. He stopped drinking alcohol at age 47; before that, he drank only occasionally. There was no notable family history of malignancy among first-degree relatives. Laryngoscopy revealed a mass in the left hypopharynx, and biopsy demonstrated squamous cell carcinoma (cT1N0M0). ^18^F-FDG PET/CT performed before referral to our department showed FDG uptake confined to the hypopharyngeal lesion, with no other abnormal FDG uptake. The patient completed definitive RT and achieved a complete endoscopic and radiological response.

During the same evaluation period, a screening colonoscopy identified a 25-mm sessile tumor in the sigmoid colon. Laparoscopic sigmoidectomy with D3 lymphadenectomy was performed first, 1 month before pancreatic surgery; pathology showed a moderately differentiated adenocarcinoma invading the muscularis propria with no nodal involvement (pT2N0M0, stage I).

**Fig. 2:**
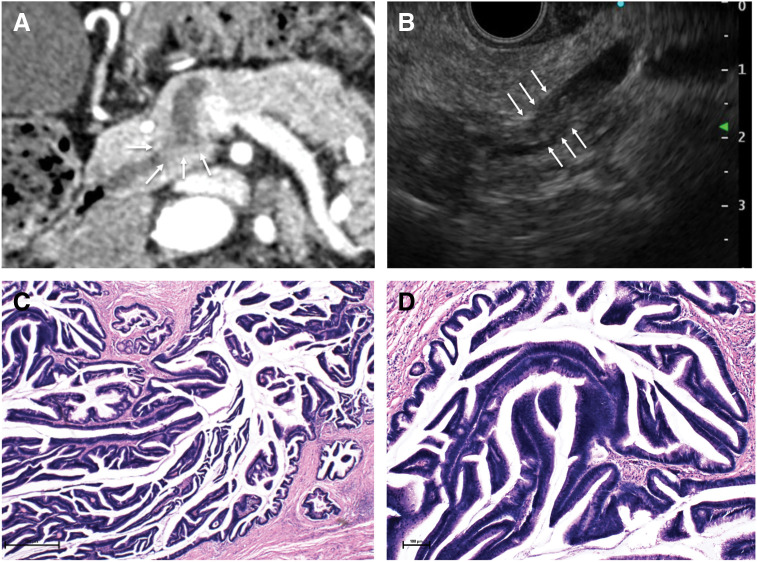
CT, EUS, and histopathological findings of IPMN. (**A**) CT revealed a 5-mm uniform dilatation of the MPD with a 15-mm enhancing mural nodule (arrows) at the pancreatic head. (**B**) EUS demonstrated a 19 × 4 mm papillary mass (arrows) in the MPD at the head of the pancreas. (**C**, **D**) Histopathology showing noninvasive IPMC (c: ×40; d: ×400). EUS, endoscopic ultrasonography; IPMC, intraductal papillary mucinous carcinoma; IPMN, intraductal papillary mucinous neoplasm; MPD, main pancreatic duct

A staging multidetector CT scan performed before RT and sigmoidectomy showed a 5-mm uniform dilatation of the main pancreatic duct and a 15-mm enhancing mural nodule at the head of the pancreas (**Fig. 2A**). Serum tumor markers (CEA 3.4 ng/mL; CA19-9 9.2 U/mL) and routine laboratory tests were unremarkable. This enhancing mural nodule met the guideline-based criteria for high-risk stigmata of IPMN,^4)^ and further pancreatic evaluation was performed. EUS revealed a 19 × 4 mm papillary lesion in the pancreatic head duct, consistent with an IPMN with high-risk features (**Fig. 2B**). Subtotal stomach-preserving pancreaticoduodenectomy was therefore performed 6 weeks after colectomy because the lesion was localized to the pancreatic head duct. Histopathology confirmed noninvasive IPMC with negative margins and lymph nodes (pTisN0M0) (**Fig. 2C** and **2D**). Postoperative recovery was uneventful, and the patient was discharged on POD 21.

At the age of 78 years, a routine surveillance CT scan detected a solitary 12-mm nodule in the right middle lobe. Thoracoscopic middle lobectomy confirmed squamous cell carcinoma with a pathological stage of IA (pT1aN0M0).

**Fig. 3:**
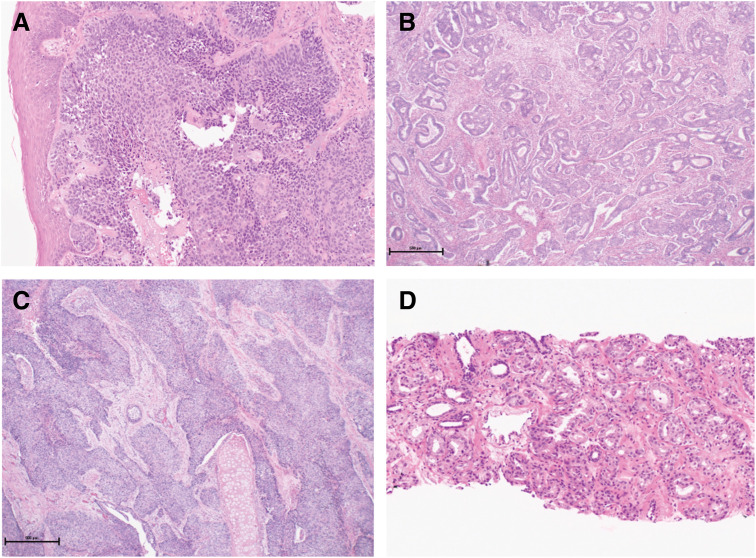
Histopathological findings of the other primary cancers. (**A**) Squamous cell carcinoma of the hypopharynx (×400). (**B**) Adenocarcinoma of the sigmoid colon (tub2 > tub1) (×40). (**C**) Squamous cell carcinoma of the lung (×400). (**D**) Acinar adenocarcinoma of the prostate (×400).

The patient remained well until age 81, when urinary hesitancy developed. The prostate-specific antigen was 16.55 ng/mL, and a multiparametric MRI showed a PI-RADS 5 lesion. A transrectal biopsy revealed acinar adenocarcinoma with a Gleason score of 4 + 3. Combined androgen blockade followed by external beam RT was administered. One year after completion of prostate RT, the patient remains asymptomatic. Histopathologic examination supported the diagnosis of mutually independent primary malignancies, without continuity from one to another (**Fig. 3A**–**3D**). Surveillance imaging of the pancreatic remnant, head and neck, chest, and colon—together with an undetectable PSA level—shows no evidence of recurrence or new malignancy.

## DISCUSSION

As life expectancy rises and diagnostic imaging improves, MPMs are encountered more frequently; however, the combination of 5 independent primary cancers in a single patient remains exceptional. MPMs were defined according to the criteria of Warren and Gates: each tumor must be malignant, distinct, and not a metastasis of another lesion.^1)^ This case involved quintuple primary cancers together with a noninvasive IPMC, and IPMN/IPMC has been reported to accompany extrapancreatic malignancies.^5,6)^ Early Japanese cohort studies reported extrapancreatic malignancies in approximately 25% of patients with IPMN, with colorectal and gastric cancers predominating.^2,3)^ According to some reports, rates of extrapancreatic malignancies in IPMN cohorts range from approximately 22% to 33%, with colorectal, breast, renal cell, prostate, and thyroid cancers being most frequently observed.^7–9)^ In our patient, colorectal cancer was identified during the initial workup, followed by lung and prostate cancers during follow-up. At the same time, whether IPMN itself increases the risk of metachronous extrapancreatic malignancies remains debated. Large multicenter and population-based studies have found that the incidence of metachronous extrapancreatic malignancies after IPMN diagnosis is not significantly higher than in the general population.^10–12)^

The etiological link between IPMN and other tumors remains speculative. Somatic *KRAS* and *GNAS* mutations—hallmarks of IPMN—are also common in colorectal carcinogenesis.^13)^ This overlap may point to shared susceptibility in some patients, but it does not readily explain hypopharyngeal and lung squamous cell carcinomas or prostate adenocarcinoma in the same individual. Therefore, in addition to possible shared biology, factors such as aging, common carcinogenic exposures, and increased detection through repeated examinations may have contributed to the pattern seen in this case. In our patient, the lung squamous cell carcinoma was considered a second primary rather than metastasis from hypopharyngeal cancer because it presented as a solitary pulmonary nodule and there was no locoregional or cervical nodal recurrence of the hypopharyngeal carcinoma. Pathologically, the tumor showed a peribronchial distribution and an intraepithelial component contiguous with the native bronchial epithelium, supporting a primary bronchogenic origin.

A search of the Japanese medical literature database (Ichushi-Web) using the keywords “IPMN” and “multiple primary cancers” identified only 5 reported cases of 4 or more primary malignancies, including the present case (**Table 1**).^14–17)^ Reports describing quintuple primary cancers are particularly limited, supporting that this presentation is very uncommon. In all reported cases, at least 1 of the concomitant malignancies involved the gastrointestinal or hepatobiliary-pancreatic system, although tumors in non-gastrointestinal organs were also observed. The timing of occurrence varied: while some patients developed all malignancies metachronously, others—including our patient—were diagnosed with multiple synchronous tumors. Several patients, including the present case, achieved long-term survival when each tumor was treated according to its stage and biological behavior.

**Table 1:** Reported cases of IPMN/IPMC associated with four or more primary malignancies in Japan

Year of report	Age	Sex	Synchronous cancers	Metachronous cancers	IPMN location	IPMN classification	Genetic evaluation (germline/MMR/MSI)	Follow-up	Outcome	Reference
2012	82	M	Bile duct cancer; gallbladder cancer	Prostate cancer	Head	Mixed	Not reported	2 years, 10 months	Alive without recurrence	^14)^
2016	73	F	Anal canal cancer	Endometrial cancer; breast cancer; pancreatic cancer	Head	Branch-duct	Not reported	9 years	Died of recurrence (9 months after anal canal cancer surgery)	^15)^
2017	70s	F	—	Gastric cancer; rectal cancer; bladder cancer	Head	Main-duct	Not reported	3 years	Alive without recurrence	^16)^
2023	59	F	—	Breast cancer; endometrial cancer; gastric cancer	Head	Branch-duct	Not reported	10 years	Died of recurrence (14 months after pancreatic cancer surgery)	^17)^
Present case	77	M	Hypopharyngeal cancer; sigmoid colon cancer	Lung cancer; prostate cancer	Head	Main-duct	Germline: declined MMR IHC: intact MSI: MSS	5 years	Alive without recurrence	This study

Synchronous cancers were defined as those diagnosed within 6 months of each other, and metachronous cancers as those diagnosed more than 6 months apart. Follow-up indicates time since IPMN diagnosis. “—” indicates none reported.

IHC, immunohistochemistry; IPMN, intraductal papillary mucinous neoplasm; MSI, microsatellite instability; MSS, microsatellite stable

The prognosis after successful treatment of noninvasive IPMC is generally favorable; however, the risk of new pancreatic cancer in the residual gland and of further extrapancreatic tumors persists for life. Our follow-up schedule therefore includes a pancreatic MRI every 6 months, a colonoscopy every year, a low-dose chest CT every year, and PSA monitoring. This follow-up plan was tailored to the patient’s prior cancers and risk profile, and it was aligned with contemporary recommendations and ongoing discussions in the literature.^4,18)^ However, the best approach to extrapancreatic surveillance in IPMN/IPMC has not been fully established.

This report has limitations. Comprehensive germline testing was declined at the patient’s request; therefore, hereditary cancer predisposition could not be fully assessed. Family history was negative for malignancy among first-degree relatives. Tumor-based screening, which is recommended in the evaluation for Lynch syndrome,^19)^ was performed for the colorectal cancer and showed intact MMR protein expression (*MLH1*, *MSH2*, *MSH6*, and *PMS2*) and MSS, making Lynch syndrome less likely. *RAS* and *BRAF* were wild-type, and p53 IHC showed an overexpression-type (mutation) pattern. In addition, molecular profiling of each tumor might have clarified the presence of common driver alterations. Nevertheless, this case suggests that clinicians managing IPMN/IPMC should remain alert to synchronous or metachronous malignancies and consider risk-adapted, long-term follow-up.

In summary, this case illustrates that favorable outcomes can be achieved with staged, patient-tailored management and careful follow-up, even when multiple primary cancers occur. Awareness of the possibility of multiple primary cancers and commitment to systematic surveillance are paramount for optimizing outcomes in this growing patient population.

## CONCLUSIONS

This rare case of quintuple primary malignancies—including noninvasive IPMC—underscores the need for careful, risk-adapted, long-term surveillance in patients with IPMN/IPMC and highlights the importance of individualized, staged management across multiple tumors.

## DECLARATIONS

### Use of artificial intelligence

ChatGPT (OpenAI) was used to assist with English language editing. The authors reviewed and edited the content and take full responsibility for the final manuscript.
